# Identification and Engineering of Transporters for Efficient Melatonin Production in *Escherichia coli*

**DOI:** 10.3389/fmicb.2022.880847

**Published:** 2022-06-20

**Authors:** Lei Yang, Sailesh Malla, Emre Özdemir, Se Hyeuk Kim, Rebecca Lennen, Hanne B. Christensen, Ulla Christensen, Lachlan J. Munro, Markus J. Herrgård, Douglas B. Kell, Bernhard Ø. Palsson

**Affiliations:** ^1^Novo Nordisk Foundation Center for Biosustainability, Technical University of Denmark, Lyngby, Denmark; ^2^Institute of Systems, Molecular and Integrative Biology, University of Liverpool, Liverpool, United Kingdom; ^3^Department of Bioengineering, University of California, San Diego, San Diego, CA, United States; ^4^Department of Pediatrics, University of California, San Diego, San Diego, CA, United States

**Keywords:** membrane, transporter, *E. coli*, inhibition, production, identification, screening, toxicity

## Abstract

Transporter discovery and engineering play an important role in cell factory development. Decreasing the intracellular concentration of the product reduces product inhibition and/or toxicity. Lowering intracellular concentrations is especially beneficial for achieving a robust strain at high titers. However, the identification of transporters for xenobiotic chemicals in the host strain is challenging. Here we present a high-throughput workflow to discover *Escherichia coli* transporters responsible for the efflux of the inhibitory xenobiotic compound melatonin. We took advantage of the Keio collection and screened about 400 transporter knockouts in the presence of a high concentration of melatonin. We found five transporters that when knocked out showed decreased tolerance to melatonin, indicating they are exporters of melatonin. We overexpressed these five genes individually in the production strain and found that one of them, *yhjV*, encoding a transporter with unknown substrates, resulted in a 27% titer increase in cultivation mimicking fed-batch fermentation. This study demonstrates how microbial cell factories can be improved through transporter identification and engineering. Further, these results lay the foundation for the scale-up of melatonin production in *E. coli*.

## Introduction

Production of chemicals (fine or/and bulk) using microbial cell factories plays a critical role in the transition to sustainable manufacturing. Among the many challenges, a major one is product inhibition, especially at high titers at the late stage of fermentation. High concentrations of the end product in the cell may inhibit enzymes of the biosynthetic pathway, slow down the reactions, and in some cases inhibit growth. This could lead to instability of the strain and cause problems when scaling up the fermentation processes (van der Hoek and Borodina, 2020; Yang et al., 2021). Engineering membrane transporters is an efficient approach to reduce the intracellular concentration of the product either by preventing the uptake or by improving efflux. This will in turn decrease cellular stresses caused by toxic products, which is also beneficial for the production pathway (Kell et al., 2015; Kell, 2019).

Identifying transporters that are responsible for the transport of the targeted compound is challenging. Predicting a transporter substrate from the existing knowledge of transporters directly is difficult, due to the complex relationship between transporter sequences and their substrates, and the fact that most transporters evolved divergently (Höglund et al., 2011).

Despite the challenges, there have been advances in the identification of transporters for a target compound. Knocking out importers of a toxic compound will render cells more resistant to this compound, which can be used as a screening tool (Lanthaler et al., 2011; Winter et al., 2014). Adaptive laboratory evolution (ALE) is an efficient way to improve tolerance against a toxic compound, and often leads to the discovery of native transporters that can be repurposed for use in production strains (Pereira et al., 2019). Genee et al. used biosensors to screen metagenomics libraries, which allowed the discovery of a novel transporter for thiamin (Genee et al., 2016). Wang et al. reported a genome-wide transporter disruption and screening workflow, where they identified transporters involved in the transport of a few xenobiotic compounds (Wang et al., 2021). Similarly, Malla et al. identified an active L-lysine exporter from the functional screening of cow gut metagenomic libraries in *Escherichia coli* where the identified lysine-specific exporter does not have any sequence homology with the previously characterized L-lysine exporters (Malla et al., 2022).

In this study, we present a workflow utilizing the existing *E. coli* Keio collection containing genome-wide single gene knockouts (Baba et al., 2006) and screening of growth inhibition to identify *E. coli* transporters that are responsible for melatonin efflux. Melatonin is an important hormone that regulates the circadian cycle of animals (Kell, 2009; Reiter et al., 2009). It is commonly used as a sleep aid and is sold as prescription medicine or as an over-the-counter dietary supplement. Melatonin is in high demand and it is currently produced by chemical synthesis. To assist the current melatonin production process with a bio-sustainable route, we have previously engineered an *E. coli* strain to produce melatonin from tryptophan at a titer of ∼2 g/L in a fermentation (Luo et al., 2020a). It has been reported that melatonin inhibits *E. coli* growth (Lopes and Sourjik, 2018), which can be a major bottleneck when scaling up the fermentation process to reach higher titers.

To address this toxicity issue, we performed a high-throughput screening of transporter knockouts to identify potential melatonin transporters. We collected a library of 394 transporter knockouts from the Keio collection (Baba et al., 2006) and screened for strains that showed altererd growth compared to the wild-type strain in the presence of high concentration of melatonin. We eventually identified five transporter knockout strains that showed impaired growth in the presence of melatonin. One of them, YhjV, is a previously unknown transporter. Furthermore, overexpression of some of the transporters by fine-tuning their expression resulted in improved titer of the melatonin production.

## Results

### Product Inhibition of the Melatonin Cell Factory

**FIGURE 1:**
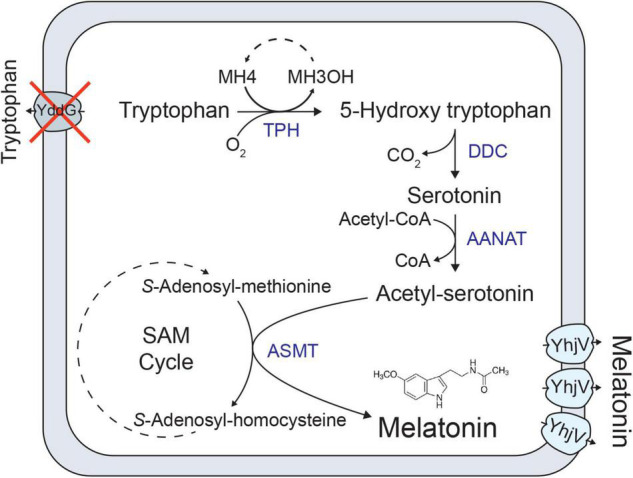
Melatonin biosynthesis pathway from tryptophan. Heterologous genes introduced into *E. coli* are indicated in blue. Cross indicates deletion of a native *E. coli* gene. Heterologous genes introducing 5-Hydroxytryptophan decarboxylase (DDC), aralkylamine N-acetyltransferase (AANAT), tryptophan hydroxylase (TPH); a pterin-4-alpha-carbinolamine dehydratase (PCD), and acetylserotonin O-methyltransferase (ASMT) expressed using constitutive promotors. Transporters related to tryptophan uptake (YddG) and melatonin efflux (YhjV) are also shown.

**FIGURE 2:**
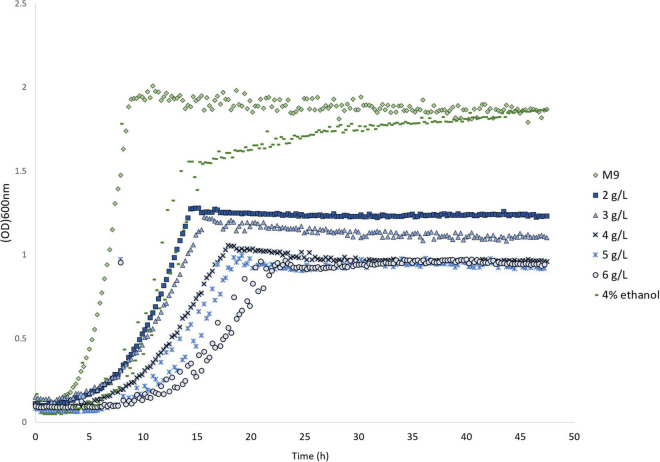
Product inhibition of the melatonin cell factory. Growth inhibition of the *E. coli* host strain HMP1741 at different concentrations of melatonin. Data represent the average of triplicates. Ethanol was added to dissolve melatonin in the media. All media with different melatonin concentrations were adjusted to contain 4% ethanol.

We have previously developed a melatonin production strain in *E. coli* (Luo et al., 2020a). In total, five heterologous enzymes were expressed in the *E. coli* BW25113 strain to produce melatonin from tryptophan (Figure 1). It is reported that low (1 mM or 232 mg/L) concentration, melatonin inhibits *E. coli* growth (Lopes and Sourjik, 2018), which agrees with our observation (Supplementary Material). We further tested the growth of an *E. coli* strain (HMP1741, a host strain for melatonin production) in the presence of various high concentrations (2–6 g/L) of melatonin in the growth medium. Melatonin was added into M9 medium supplemented with 0.2% glucose and the growth curves were monitored. As shown in Figure 2, compared to the control, the final biomass decreased ∼30% at 2 g/L melatonin, and further increasing melatonin concentration to 6 g/L decreases biomass yield by an additional 30%. Note that the control condition was M9 medium containing 4% ethanol, as ethanol was used as a solvent to dissolve melatonin. The data confirmed that melatonin severely inhibits the growth of the *E. coli* strain at concentrations above 2 g/L. This result allows efficient screening of transporter knockout libraries through differed growth profiles.

### Identification of Melatonin Transporters

**FIGURE 3:**
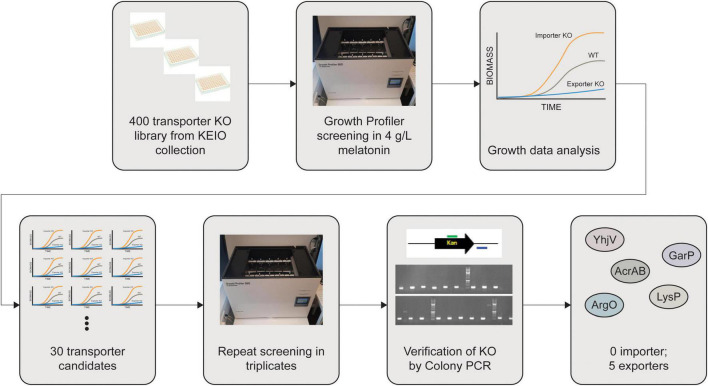
Workflow for identification of melatonin transporters. About 400 Keio knockouts with transporter genes deleted were screened in the presence of high concentrations (>2 g/L) of melatonin. Gene deletions were further confirmed using colony PCR. Five knockouts giving rise to decreased melatonin tolerance are considered to play major roles in melatonin efflux.

Since melatonin toxicity is one of the major bottlenecks during its fermentative production, we sought to mitigate melatonin inhibition by lowering the intracellular concentration of melatonin. We designed a workflow (Figure 3) to identify transporters capable of exporting melatonin in *E. coli*. It is anticipated that a melatonin importer deletion strain will grow better due to the reduced uptake of melatonin. Conversely, a melatonin exporter deletion strain can exhibit growth defects due to higher intracellular melatonin accumulation.

We have previously gathered an *E. coli* transporter knockout library (Jindal et al., 2019) predominantly selected from TransporterDB (Elbourne et al., 2017). Some knockout strains were missing from the Keio collection, resulting in a library of 522 transporter knockouts, of which 88% overlap with TransporterDB *E. coli* transporters. For proteins that are known to form protein complexes to be functional, we only included the knockout strain for a single subunit. Our final library consisted of a total 394 knockout strains from the Keio collection (Baba et al., 2006; Yamamoto et al., 2009). The list of these strains is given in the Supplementary Material. We analyzed the growth of these strains in the presence of 4 g/L of melatonin using plate-based high throughput growth screening (see section “Materials and Methods”). In such screening, higher growth rates and shorter lag phases compared to the wild-type strain can be expected in case a melatonin importer is knocked out, whereas a lower growth rate and a longer lag phase can be expected if a melatonin exporter is deleted (Figure 3).

From the results of the first screening round (in singlet), 30 candidate transporter targets were identified. In a second round, the growth test was repeated for those selected 30 candidates in triplicates. In parallel, we also performed colony PCR to confirm the knockout strain. Two of the 30 strains were excluded because they did not contain the expected gene knockouts. From the second round of screening, we identified five transporters responsible for melatonin tolerance; YhjV, GarP, ArgO, AcrB, and LysP (Table 1). However, we did not identify any importers. Despite being a xenobiotic, melatonin is likely imported by numerous promiscuous nutrient influx transporters, due to its structural similarity to tryptophan. Previous modeling has shown that in the presence of equilibrative transporters a concentrative uptake transporter and an efflux transporter for a given substrate, alterations of influx transporter function do not significantly alter the intracellular concentration of the substrate (Mendes et al., 2020). The existence of numerous unspecific importers that can uptake melatonin is the likely reason that we were unable to identify a single importer knockout in this assay.

**TABLE 1:** The five transporters identified to be responsible for melatonin export.

Name (gene)	Description	UniProtKB reference
YhjV (*yhjV*)	Putative amino acid transporter	UniProtKB – P37660 (YHJV_ECOLI)
GarP (*garP*)	Galactarate/glucarate/glycerate transporter	UniProtKB – B1LFM8 (B1LFM8_ECOSM)
ArgO (*argO*)	L-arginine efflux transporter	UniProtKB – P11667 (ARGO_ECOLI)
LysP (*lysP*)	lysine:H + symporter	UniProtKB – P25737 (LYSP_ECOLI)
AcrA (*acrA*)	Multidrug efflux pump subunit	UniProtKB – P0AE06 (ACRA_ECOLI)
AcrB (*acrB*)	Multidrug efflux pump subunit	UniProtKB – P31224 (ACRB_ECOLI)

**FIGURE 4:**
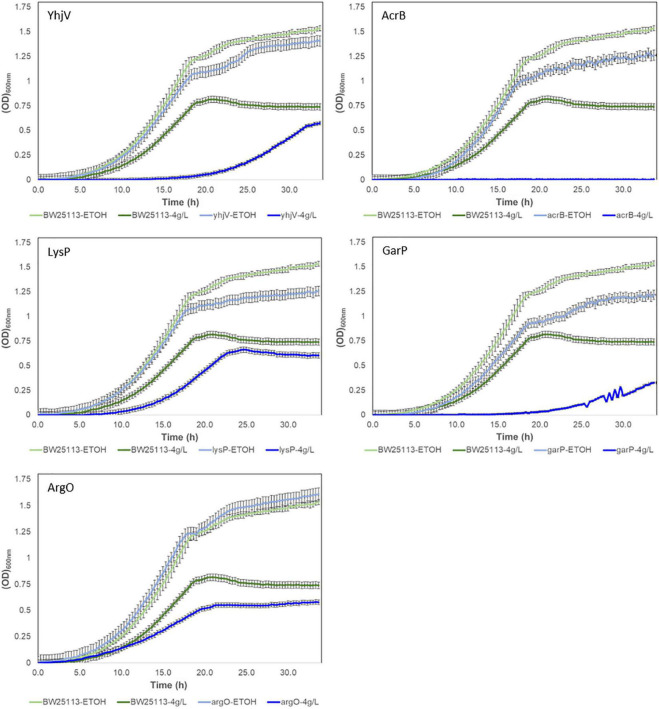
Growth of Keio transporter knockouts in the presence of 4 g/L of melatonin in M9 glucose media. BW25113 (wild-type strain of the Keio knockouts) was used as a control strain. All strains were cultivated in the presence of 4% melatonin and 4% ethanol (−4 g/L). The control condition is the medium supplemented with only 4% ethanol (-ETOH).

Growth curves of the five selected transporter candidate deletion strains in the presence of 4 g/L of melatonin are shown in Figure 4. All five strains have decreased growth rates compared to the wild-type. The most prominent effect resulted from the *acrB gene*, whose deletion eliminates growth. Two transporter gene deletions, *yhjV* and *garP*, increased lag time and reduced growth rates. The *lysP* and *argO* deletion strains also showed reduced growth rates but no significant difference in lag-phase compared to the wild-type control strain (Figure 4). In summary, we observed that strains lacking any one of these five transporters are not able to maintain the same level of melatonin tolerance as compared to the control strain. This strongly suggests that these strains have a higher melatonin accumulation and these transporters are critical for the efflux of intracellular melatonin.

### Overexpression of Melatonin Transporters to Improve Melatonin Production

**FIGURE 5:**
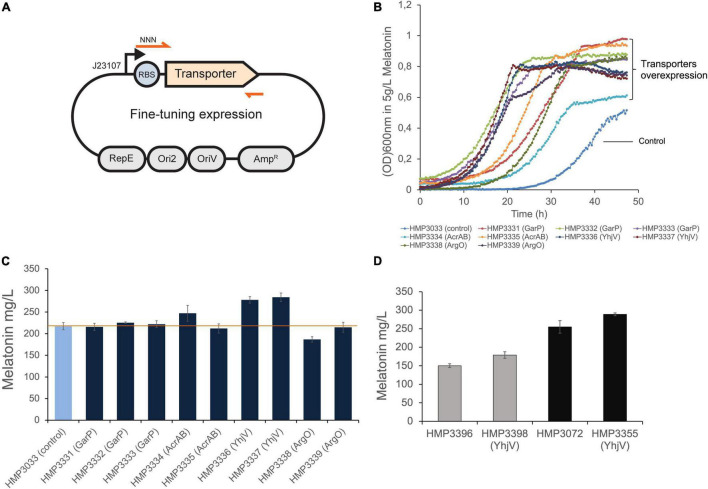
Overexpression of transporters and their impact on melatonin production. **(A)** The transporter genes were cloned in a low copy plasmid backbone (Womble and Rownd, 1987) with RBS containing degenerate sequences under the control of the J23107 promoter. **(B)** The selected resulting strains with AcrAB, YhjV, GarP and ArgO overexpression gave rise to increased tolerance to 5 g/L melatonin. **(C)** The selected strains from **(B)** were further tested for melatonin production in small-scale assays. Extra-cellular melatonin was measured in this assay. The 2 YhjV overexpression strains showed over 20% improvement in titer. **(D)** The transporter plasmid in HMP3337 was extracted and transformed into 2 other melatonin production strains, offering about 13 and 19% titer increase, respectively. Presented data are mean ± s.d. (*N* = 3). Promoter and RBS sequences are listed in Supplementary Material.

To implement the newly discovered transporters for melatonin production, we overexpressed these genes to increase melatonin tolerance in *E. coli*. Each transporter gene was cloned under the control of a weak constitutive promoter in a low copy number plasmid (Figure 5A). Furthermore, to ensure sufficient expression level without causing too much cellular stress, we fine-tuned the translation efficiency of each transporter. We designed degenerate sequences in ribosome binding sites (RBS) of each transporter gene, which normally give rise to a range of translation initiation rates of the target genes (Salis et al., 2009; Figure 5A, Materials and Methods). The transporter plasmid libraries were transformed into a melatonin production strain. The strains containing RBS libraries were cultivated in the 5 g/L of melatonin over 16 h to enrich the optimal RBS sequences. In the case of AcrA and AcrB that form protein complexes to function, we expressed both genes in an operon.

The enriched populations with RBS libraries were spread in LB-agar (Amp) to obtain single colonies. For each transporter, about 9–16 individual isolates were selected to test their ability melatonin production using a small-scale production assay (Materials and Methods). Strains with optimized expression of GarP, AcrAB, YhjV, or ArgO showed improved melatonin titers, whereas LysP did not show significant improvement with any of the selected RBS sequences (Supplementary Material). We further selected 2–3 colonies for each transporter GarP, AcrAB, YhjV, or ArgO. We confirmed their melatonin tolerance (Figure 5B) and repeated the production assay in triplicates (Figure 5C). One construct of AcrAB overexpression offered a small increase in melatonin titer. The orphan transporter, YhjV, increased melatonin titer by ∼27% when overexpressed. This strain can be selected as the lead strain for further process optimization.

We also transformed the YhjV overexpression plasmid pHM635 from HMP3337 into other melatonin-producing strains to evaluate its effect. Two strains HMP3396 with dihydromonapterin reductase FolM overexpression (Lin et al., 2014) and HMP3072 containing FolM overexpression and anthranilate synthase subunit TrpE mutant S40F (Caligiuri and Bauerle, 1991) were tested (Table 2). As shown in Figure 5D, YhjV overexpression resulted in 13 and 19% increase in melatonin titer for strain HMP3396 and HMP3072, respectively. This suggests that YhjV overexpression can be a general approach to improve melatonin production in *E. coli*.

**TABLE 2:** Strains and plasmids used in this study.

Strain ID	Genotypes*	Source/Origin
BW25113	F-, λ-, *Δ(araBAD)567, ΔlacZ4787(:rrnB3), Δ(rhaBAD)568, rph-1, hsdR514*	Baba et al., 2006
HMP1741	BW25113 Δ*tnaA* Δ*trpR* FolE(T198I) ΔPfolE:PJ23100 Δ*fhuA* Ptrc:ddc-aanat	This work
HMP2993	BW25113 Δ*tnaA* Δ*trpR* FolE(T198I) ΔPfolE:PJ23100 Δ*fhuA* P2:*ddc* J23101:*aanat*	This work
HMP3033	HMP2993 + pHM345	This work
HMP3331	HMP2993 + pHM345 + pHM629	This work
HMP3332	HMP2993 + pHM345 + pHM630	This work
HMP3333	HMP2993 + pHM345 + pHM631	This work
HMP3334	HMP2993 + pHM345 + pHM632	This work
HMP3335	HMP2993 + pHM345 + pHM633	This work
HMP3336	HMP2993 + pHM345 + pHM634	This work
HMP3337	HMP2993 + pHM345 + pHM635	This work
HMP3338	HMP2993 + pHM345 + pHM636	This work
HMP3339	HMP2993 + pHM345 + pHM637	This work
HMP3396	HMP2993 J23107:*folM* TrpE(S40F)	This work
HMP3398	HMP3396 + pHM635	This work
HMP3072	HMP2993 J23107:*folM*	This work
HMP3355	HMP3427 + pHM635	This work
HMP3403	BW25113 Δ*tnaA* Δ*trpR* FolE(T198I) ΔPfolE:PJ23100 Δ*fhuA* Δ*gstA* + pHM402	This work
		This work
HMP3404	HMP3403 + pHM635	This work

**Plasmid ID**	**Genotype**	**Source/Origin**

pHM345	J23107:*tph-pcd-asmt Kan^R^*	This work
pHM402	J23107:*tph-pcd Amp*^R^**	This work
pHM629-631^#^	J23107:*garP*	This work
pHM632, pHM633	J23107:*acrA*	This work
pHM634, pHM635	J23107:*yhjV*	This work
pHM636, pHM637	J23107:*argO*	This work

*ΔPfolE:PJ23100 indicates promoter of folE gene is changed to a constitutive promoter J23100. ^#^Plasmids pHM629-631 have the same promoter and different RBS sequences. FolE(T198I) indicates a mutation causing an amino acid change T198I, which was previously characterized (Luo et al., 2020b). See Supplementary Material for details.

### Overexpression of Melatonin Transporters to Improve 5-Hydroxytryptophan Production

**FIGURE 6:**
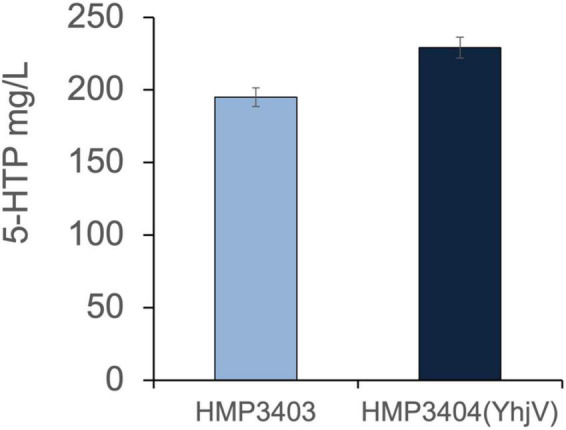
Overexpression of YhjV increased 5-HTP production. Production of 5-HTP was tested in small-scale assays (Materials and Methods). Strain HMP3404 with YhjV overexpression offers an increased 5-HTP production compared to HMP3403 as measured in the supernatant. Presented data are mean ± s.d. (*N* = 3).

Given that 5-Hydroxytryptophan (5-HTP) is also an important tryptophan derivative and an intermediate of melatonin biosynthesis (Figure 1), we also tested the effect of YhjV transporter overexpression in 5-HTP production. The 5-HTP production strain (HMP3403) was generated by overexpressing tryptophan dehydrogenase (TpH) and pterin-4-alpha-carbinolamine dehydratase (Pcd). The strain utilized O_2_ and tetrahydromonapterin (MH_4_) as co-factors for TpH as described in a previous publication (Luo et al., 2020b). We transformed the plasmid (pH635) with YhjV overexpression into HMP3403, and the resulting strain HMP3404 improved 5-HTP titers by 15% compared to the control HMP3403 (Figure 6) in a small-scale production assay (see section “Materials and Methods”).

## Discussion

Melatonin is an important tryptophan derivative, which is used as a nutraceutical supplement and prescription medication. We have previously reported an *E. coli* cell factory producing melatonin by feeding tryptophan (Luo et al., 2020b). One of the steps that improved the production was knocking out a known tryptophan exporter gene, *yddG*, which allows for maintaining a high intracellular tryptophan concentration (Figure 1). Here we demonstrated the discovery and overexpression of a previously unknown melatonin transporter to further enhance melatonin titers. Figure 1 illustrates how deleting *yddG* and elevated expression of *yhjV* boosts melatonin production.

Transporter discovery and their manipulation are difficult. There are very few examples that membrane transporter can be identified through a substrate similarity search (Kurgan et al., 2019; Kell, 2021). The challenges and advances have been recently reviewed (Kell et al., 2015; Borodina, 2019; Kell, 2019; van der Hoek and Borodina, 2020). Here we developed a workflow using the existing knockout library to screen for transporters that could mitigate product toxicity. Single-gene knockout libraries are important resources to study gene functions. Such strain libraries have been constructed and made available for many model organisms, including *E. coli, Bacillus* spp, and yeast (Baba et al., 2006; Yamamoto et al., 2009; Giaever and Nislow, 2014; Koo et al., 2017). Screening such libraries using differentiated growth or biosensors is a simple and high-throughput way to identify transporters. This workflow can also be combined with ALE to further improve tolerance to toxic products.

Among the newly discovered transporters in this study, YhjV is annotated as an uncharacterized transporter, belonging to the hydroxy/Aromatic Amino Acid Permease (HAAAP) Family (Saier et al., 2014). There is no function associated with this transporter according to EcoCyc (Keseler et al., 2021), except that *yhjV* deletion has higher sensitivity toward radiation (Sargentini et al., 2016). YhjV was found to be responsible (in part) for the uptake of a fluorescence dye, SYBR Green (Jindal et al., 2019). Since SYBR green is a synthetic compound, the native function of this transporter was still to be revealed. Our discovery about YhjV as a melatonin transport gives an important clue to the function of this transporter. Melatonin is produced in human bodies by not only the pineal gland but also other glands/cells, including the gastrointestinal tract (Tordjman et al., 2017). Since *E. coli* is a commensal bacterium isolated from human guts, it may have evolved transporters for melatonin uptake or efflux. Interestingly, Lopes and Sourjik found that melatonin is a chemorepellent for *E. coli* MG1655. *E. coli* can sense melatonin and react by repelling this chemical using its chemotaxis systems (Lopes and Sourjik, 2018). It is reasonable to speculate that melatonin transport is a native function of YhjV in *E. coli*, which evolved in the gut to reduce melatonin toxicity. Further investigation of other substrates of YhjV is ongoing to better describe this transporter.

The other four transporters that were found to be important for melatonin tolerance have better annotations as compared to YhjV. AcrAB is a well-known multidrug efflux pump. Predictably, AcrB knockout has a growth defect in high concentrations of melatonin. GarP is a member of the Anion: Cation Symporter (ACS) Family, involved in the uptake of diacid sugars, such as galactarate and D-glucarate. ArgO is an arginine exporter and LysP is a lysine-specific importer. It is not clear why overexpressing these transporters did not result in a significant improvement in melatonin titer despite the increase in melatonin tolerance in the corresponding knockout strains. One explanation is that some transporters have other functions, and thus expression of this transporter gene through a constitutive promoter may cause pleiotropic effects of inhibiting growth and production. For example, ArgO overexpression might have a negative impact on maintaining the intracellular arginine; AcrAB, which forms a complex with TolC, has a wide range of substrates. In contrast, YhjV might have a relatively narrow range of substrates, and hence overexpressing it has a more specific effect. It is worth noting that YhjV is a predicted permease, where the gradient of the substrate concentration plays an important role in its function. It will be interesting to investigate how the strain harboring the YhjV expression plasmid behaves in strains producing different levels of melatonin.

In conclusion, we have demonstrated a successful example of applying transporter discovery and manipulation of transporter expression to enhance the production of melatonin in *E. coli*. We identified five transporters responsible for melatonin export. One of them, YhjV, is an unknown transporter, filling a knowledge gap in *E. coli* functional genomics. Overexpression of these transporters enhanced melatonin tolerance dramatically. The strain with YhjV overexpression yielded an improved titer in small-scale cultivation systems mimicking fed-batch fermentation. It has a similar benefit in a 5-HTP producing strain too. This work has paved the way for producing melatonin at a commercially feasible level using microbial hosts. We believe that the workflow described here is an important step toward better control of the intracellular concentrations of substrates, intermediates, and products during cell factory development, and this workflow can be generally applied for finding active metabolite-specific transporters.

## Materials and Methods

### Strains and Plasmids

Melatonin production strains were derived from BW25113 (Figure 1). To verify gene deletions in Keio collection strains, colony PCR was performed using a primer located in the kanamycin-resistant cassette (ATATTGCTGAAGAGCTTGGC) and a reverse primer located downstream of the targeted gene. A correct knockout strain generates a DNA band in electrophoresis. If no bands were generated, the knockout strains were excluded from the following procedures.

RBS sequence containing degenerate sequences tcttaatcatgcnnkggannkttaacttt (k = g/t) was cloned upstream of the coding sequence of each gene. The plasmid was constructed using USER cloning method (Geu-Flores et al., 2007). The plasmid libraries were transformed into strain HMP2993 (Table 2) together with pHM345 containing some pathway genes and cultured in the presence of 5 g/L melatonin.

### Melatonin Inhibition Growth Tests

For growth tests, *E. coli* strains were inoculated into 400 μl of M9 + 0.2% glucose media supplemented with required antibiotics in a 96 deep well-plate Then, the plate was incubated at 30°C at 300 rpm overnight (incubated for 18–24 h). The following day, the saturated pre-cultures were diluted 100 fold into 300 μl of M9 + 0.2% glucose media (and antibiotics as required) supplemented with desired concentrations of melatonin in a 96 well MTP plate. Melatonin was purchased from Sigma-Aldrich (>98%). All cultures were protected from light to avoid melatonin degradation (Moussaoui and Bendriss, 2014). Melatonin stock solution was prepared by dissolving 170 g/L melatonin in 75% ethanol. For testing growth inhibition of various melatonin, all cultures containing different concentrations of melatonin were adjusted to have 4% ethanol (Figure 2). Cells growing in M9 + 0.2% glucose media containing 4% ethanol were used as a control. For screening of Keio knockouts collection, 4 g/L melatonin was used. The wild-type strain *E. coli* BW25113 was used as a control. For testing the RBS libraries of all transporter genes, screening was performed at 5 g/L of melatonin. Culture plates were then incubated in Growth Profiler (Enzyscreen, Heemstede, the Netherlands) at 30°C and 250 RPM for 48 h with constant monitoring of the growth by taking pictures every 20 min. The image data was converted into digital OD600 nm value using the software GP960Viewer version 1.0.0.4 (Enzyscreen, Heemstede, the Netherlands). Data analysis was done using a CROISSANCE package (Schöning et al., 2016).

### Small-Scale Production Assay

To measure the production of melatonin, bacterial cells were cultivated in deep-well plates in glucose slow-release (GSR) medium mimicking fed-batch fermentation at 30°C. 1 L of GSR medium contains 15 g of Maltodextrin (dextrose equivalent 4.0–7.0, Sigma-Aldrich), 200U of Amyloglucosidase (Sigma-Aldrich, ∼70 U/mg) for glucose release, 40 g of MES monohydrate (Sigma-Aldrich), 1.2 g of K_2_HPO_4_, 7 g of Ammonium sulfate, 120 mg of Sodium citrate, 8 mg of ZnCl_2_, 12 mg of FeSO_4_⋅7H_2_O, 9 uM of CaCl_2_, 12.5 mM of MgSO_4_⋅7H_2_O, as well as trace elements and vitamins. The media pH was adjusted to 6.4. The medium was supplemented with 500 mg/L tryptophan, 50 mg/L kanamycin, and 100 mg/L ampicillin. The bacterial cells were cultivated at 30°C in M9 medium supplemented with 0.5% glucose for 24 h. The precultures were diluted 100 times into the GSR medium and cultured at 30°C. After 24 h, the supernatant was collected by filtering the broth with 0.2 μm filters (Pall, New York, United States). The concentration of melatonin in the supernatant was determined by HPLC as previously described (Luo et al., 2019).

## Data Availability Statement

The original contributions presented in this study are included in the article/Supplementary Material, further inquiries can be directed to the corresponding author/s.

## Author Contributions

LY, RL, DK, and BP conceived the study. SM, LY, SK, UC, HC, and LM performed the experiments. SM, EÖ, SK, RL, and LY analyzed data. LY and SM drafted the manuscript and the other authors contributed to the writing. LY, MH, DK, and BP managed the project. All authors contributed to the article and approved the submitted version.

## Conflict of Interest

DK, LY, and SM are inventors of the following patent application: WO/2020/187739. 2020. The remaining authors declare that the research was conducted in the absence of any commercial or financial relationships that could be construed as a potential conflict of interest.

## Publisher’s Note

All claims expressed in this article are solely those of the authors and do not necessarily represent those of their affiliated organizations, or those of the publisher, the editors and the reviewers. Any product that may be evaluated in this article, or claim that may be made by its manufacturer, is not guaranteed or endorsed by the publisher.
