# Supplementary material for: Identification and Engineering of Transporters for Efficient Melatonin Production in Escherichia coli
Source: Front Microbiol. 2022 Jun 20;13:880847. doi: 10.3389/fmicb.2022.880847 (PMC9251470; doi:10.3389/fmicb.2022.880847)
Supplement: Supplementary file 2 [file Data_Sheet_1.pdf]

Supplementary Material

Identification and engineering of transporters for efficient melatonin production in *Escherichia coli*

Lei Yang[1#*], Sailesh Malla[1#], Emre Özdemir[1], Se Hyeuk Kim[1], Rebecca Lennen[1], Hanne B. Christensen[1], Ulla Christensen[1], Lachlan J. Munro[1], Markus J. Herrgård[1], Douglas B. Kell[1,2*], Bernhard Ø. Palsson[1,3,4]

1 Novo Nordisk Foundation Center for Biosustainability, Technical University of Denmark, Kongens Lyngby, Denmark,
2 Institute of Systems, Molecular and Integrative Biology, University of Liverpool, Liverpool, United Kingdom,
3 Department of Bioengineering, University of California, San Diego, La Jolla, California, United States,
4 Department of Pediatrics, University of California, San Diego, La Jolla, California, United States.

# These authors have equal contribution
* Corresponding authors. Lei Yang (leiya@biosustain.dtu.dk), Douglas B. Kell (Douglas.Kell@liverpool.ac.uk)

Supplementary tables

Table S1. Transporter knockout collection used in this study
Attached an excel sheet.

Table S2. Promoter sequences used in this study

Promoter	Sequence
Ptrc*	TTGACAATTAATCATCCGGCTCGTATAATGTGTGGA
P2[#]	AAAAAGAGTATTGACTTCGCATCTTTTTGTACCTATAATGTGTGGA
J23101[*]	TTTACAGCTAGCTCAGTCCTAGGTATTATGCTAGC
J23107[*]	TTTACGGCTAGCTCAGCCCTAGGTATTATGCTAGC
J23100[*]	TTGACGGCTAGCTCAGTCCTAGGTACAGTGCTAGC

* Anderson, C. https://parts.igem.org/Promoters/Catalog/Constitutive. J23107 is weaker than J23100 and J23101 according to this study.

Mutalik, V., Guimaraes, J., Cambray, G. *et al*. (2013). Precise and reliable gene expression via standard transcription and translation initiation elements. Nat Methods 10, 354–360. https://doi.org/10.1038/nmeth.2404

Table S3. RBS sequences[#] used in this study for transporter gene expression

Alias (Figure S1)	Strain ID (Figure 5)	Plasmid ID	Transporter gene	RBS sequence
HMP3280_3	HMP3331	pHM629	*garP*	tcttaatcatgcggtggaacgttaactttATG
HMP3280_15	HMP3332	pHM630	*garP**	tcttaatcatgcgggggagtgttaactttATG
HMP3280_16	HMP3333	pHM631	garP*	tcttaatcatgcgggggagtgttaactttATG
HMP3281_2	HMP3334	pHM632	*acrA_acrB*	tcttaatcatgccttggagggttaactttATG
HMP3281_14	HMP3335	pHM633	*acrA_acrB*	tcttaatcatgcgttggaggattaactttATG
HMP3282_8	HMP3336	pHM634	*yhjV*	tcttaatcatgccggggacggttaactttATG
HMP3282_11	HMP3337	pHM635	*yhjV*	tcttaatcatgccggggacggttaactttATG
HMP3284_6	HMP3338	pHM636	*argO*	tcttaatcatgcatgggaagtttaactttATG
HMP3284_12	HMP3339	pHM637	*argO*	tcttaatcatgctggggagggttaactttATG

[#]In order to obtain a range of protein expression levels, we designed a degenerate RBS sequence library: tcttaatcatgcnnkggannkttaacttt (k=g/t). For all the transporter genes, these degenerate sequences are expected to result in ranges of 10^2- 10^5 or 10^1-10^4 in translation initiation rates, as predicted by the RBS library calculator (www.denovodna.com/)[¤]. The optimized RBS sequence is shown in colors. Transcription start sites are indicated in capital letters. * GarP has an early stop codon at amino acid R135 in pHM630 and pHM631. The promoter, RBS, and transporter coding sequences turned out to be the same in pHM630 and pHM631, as well as pHM634 and pHM635.

[¤]Salis, H., Mirsky, E. & Voigt, C. Automated design of synthetic ribosome binding sites to control protein expression. Nat Biotechnol 27, 946–950 (2009). https://doi.org/10.1038/nbt.1568

Supplementary figures

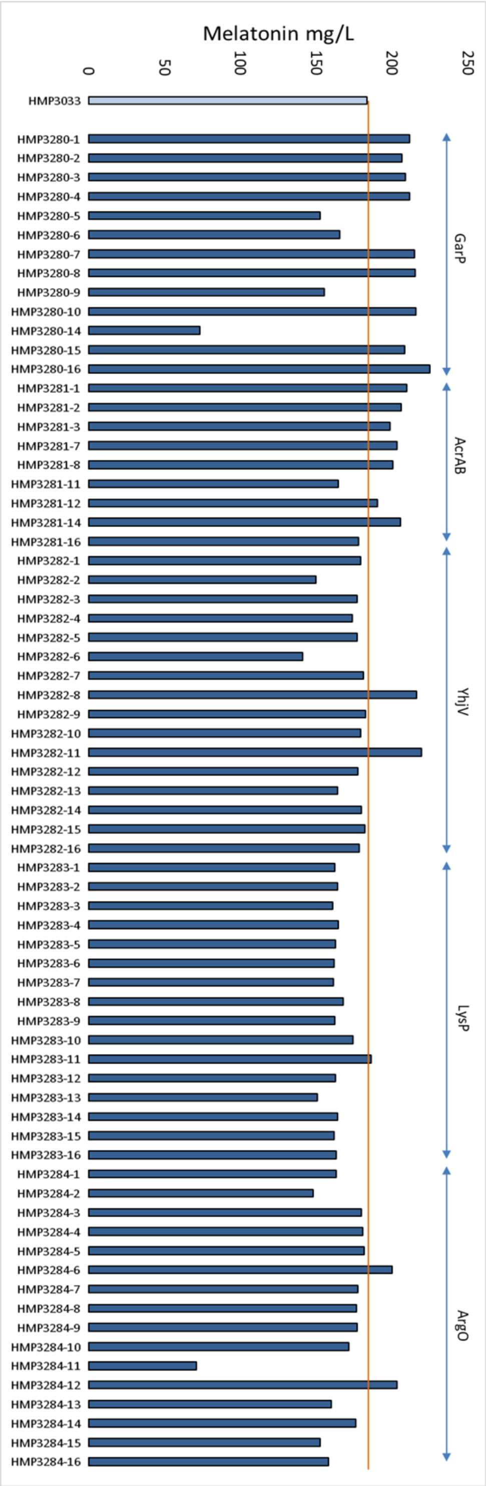

Figure S1. Melatonin production of strains with the 5 transporters overexpressed. After enrichment in 5 g/L of melatonin, 9-16 colonies were picked for each transporter and tested production in a small-scale assay. No improvement was observed in any colonies for LysP overexpression.

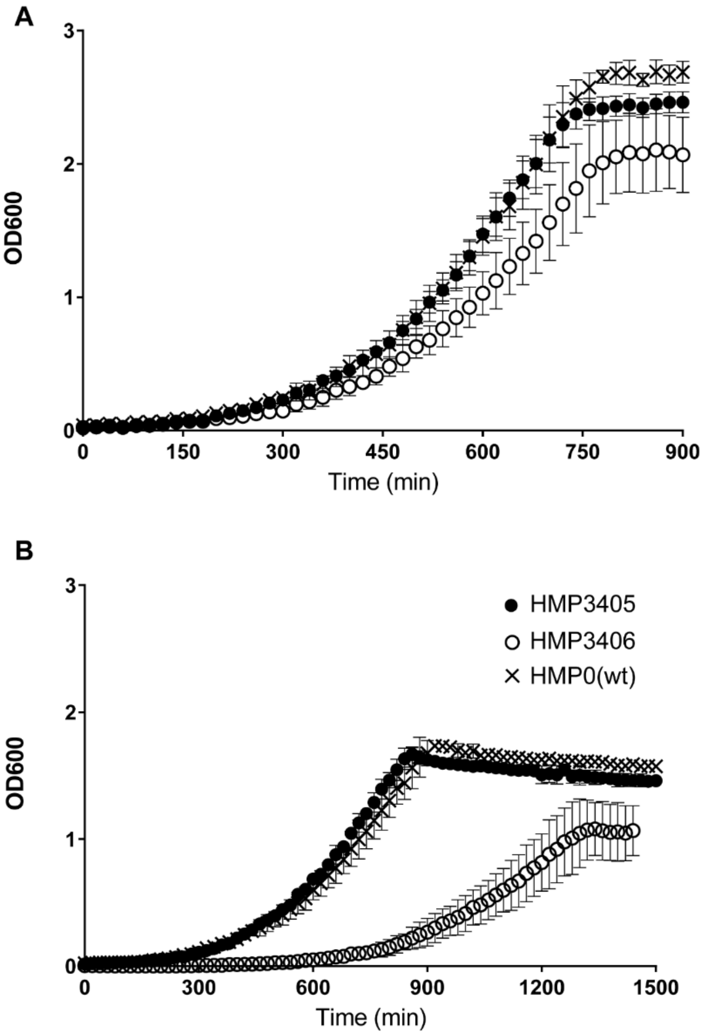

HMP3405	BW25113 Δ*yhjV*
HMP3406	HMP3405 + pHM635

Figure S2. Compensation of growth defect of transporter knockout strain by the transporter overexpression plasmid. Growth profiles of the wild-type strain (HMP0), *yhjV* deletion (HMP3405) and *yhjV* knockout strain with *yhjV* complemented in the plasmid pHM635 (HMP3406), in the presence of 4% ethanol (A) and 4 g/L melatonin + 4% ethanol (B). This confirms that a plasmid copy of *yhjV* gene is able to recover the growth defect of *yhjV* deletion in HMP3405. Also the growth defect of yhjV delection is not caused by unexpected off-target mutation of the strain. Presented data are mean ± s.d. (N =3).

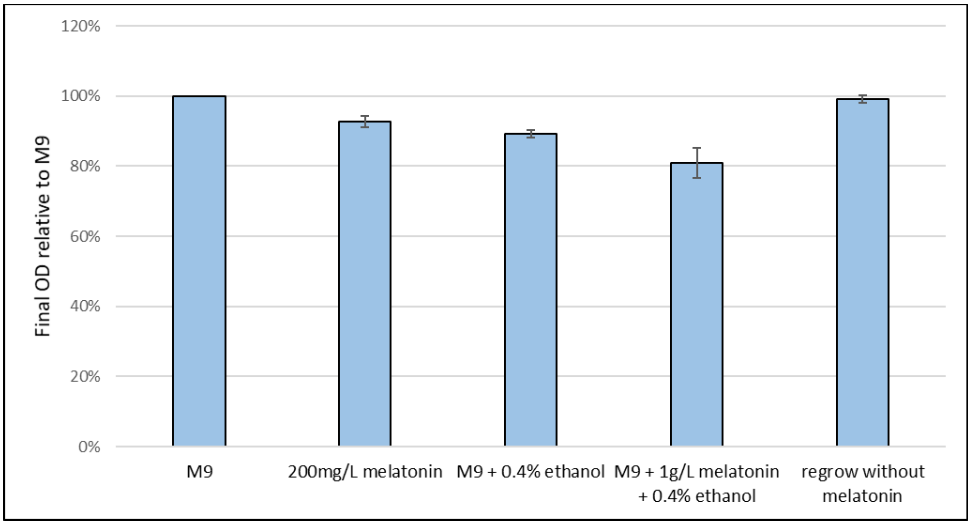

Figure S3. Melatonin inhibition at low melatonin concentrations. Strain HMP1740 showed about 7-8% reduction in final biomass yield when 200mg/L or 1g/L melatonin was added compared to the controls (M9 or M9+0.4% ethanol, respectively). The cultivation was performed in deep-well plates at 30°C with shaking (225rpm). OD(600nm) was measured after 14h incubation. The cultures containing 1g/L melatonin were diluted 100 times in a fresh M9 medium. The regrown cultures reached the same OD as M9+glucose without melatonin, indicating melatonin inhibition is reversible. Presented data are mean ± s.d. (N =3). The M9 medium contains 0.2% glucose as described in Materials and Methods.

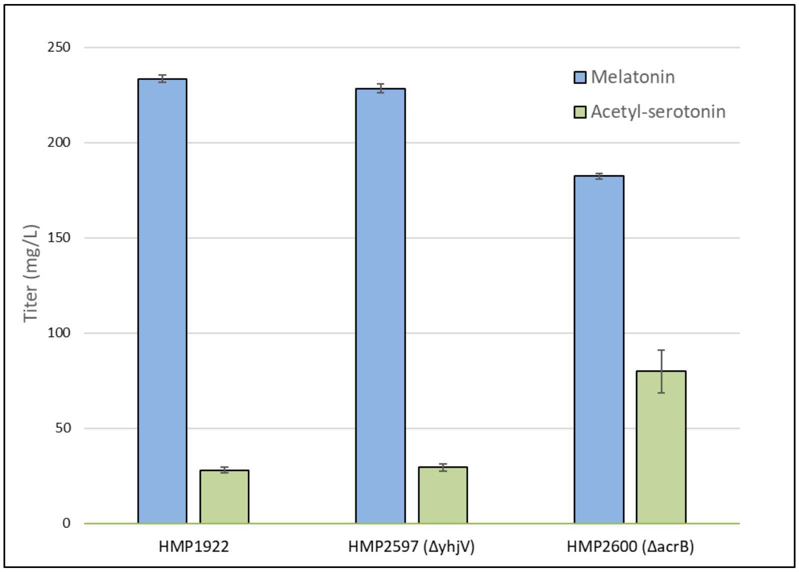

HMP1922	HMP1741+pHM151
HMP2597	HMP1741 Δ*yhjV* + pHM151

HMP2600	HMP1741 Δ*acrB* + pHM151
pHM151	p15A_J23107::*tph-pcd-asmt Amp*^{R}

Figure S4. Impact of knockout of chromosomal copies of *yhjV* and *acrB* genes in melatonin production. Deletion of *yhjV* gene in the genome leads to only 3% ($p<0.05$) decrease in the melatonin titer (Materials and Methods), probably due to compensation of other transporters and low toxicity at this melatonin concentration. Presented data are mean ± s.d. (N =3). Knockout of *acrB* gene results in a much lower melatonin titer and higher accumulation of the intermediate acetyl-serotonin (Figure 1). This suggests that AcrAB plays a bigger role in melatonin efflux, which agrees with the dramatic growth defect of the *acrB* knockout strain in melatonin shown in Figure 4.
